# D-Glucose-Induced Cytotoxic, Genotoxic, and Apoptotic Effects on Human Breast Adenocarcinoma (MCF-7) Cells

**DOI:** 10.4172/1948-5956.1000265

**Published:** 2014-04-25

**Authors:** Christine K Tchounwou, Clement G Yedjou, Ibrahim Farah, Paul B Tchounwou

**Affiliations:** Cellomics and Toxicogenomics Research Laboratory, NIH-Center for Environmental Health, College of Science, Engineering and Technology, Jackson State University, USA

**Keywords:** D-glucose, MCF-7 cells, Cytotoxic, DNA damage, Apoptosis

## Abstract

**Introduction:**

Glucose is a simple sugar that plays an important role in energy production in biological systems. However, it has been linked to many long-term health problems including the risk of heart disease and stroke, erectile dysfunction in men and pregnancy complications in women, and damage to the kidneys, nerves, eye and vision. Also, the underlying mechanisms of diabetic complications are poorly understood.

**Methods:**

In the present study, D-glucose-induced cytotoxic, genotoxic, and apoptotic effects were studied using MCF-7 cells as an *in vitro* test model. Cell viability was determined by MTT assay. Genotoxic damage was tested by the means of alkaline single cell gel electrophoresis (Comet) assay. Cell apoptosis was measured by flow cytometry assessment (Annexin-V/PI assay).

**Results:**

The results of MTT assay indicated that D-glucose significantly reduces the viability of MCF-7 cells in a dose and time-dependent manner. Similar trend was obtained with the trypan blue exclusion test. Data obtained from the Comet assay indicated that D-glucose causes DNA damage in MCF-7 cells in a dose-dependent manner. The flow cytometry assessment (Annexin V FITC/PI) showed a strong dose-response relationship between D-glucose exposure and annexin V positive MCF-7 cells undergoing early apoptosis.

**Conclusion:**

Taking together, these data provide clear evidence that D-glucose induces cytotoxic, genotoxic, and apoptotic effects on MCF-7 cells. This finding represents the basis for further studies addressing the pathophysiological mechanisms of action of glucose overdose.

## Introduction

Glucose (C_6_H_12_O_6_) also known as D-glucose, dextrose, or grape sugar is a simple monosaccharide found in plants. It is the major source of energy for most cells of the body systems. Glucose, fructose, and galactose are three dietary monosaccharides that are absorbed directly into the bloodstream during digestion. Although glucose plays an important role in energy production in our body, a previous report indicated that intermittent high glucose induces apoptosis due to activation of cellular oxidative stress in human umbilical vein endothelial cells [1]. Reactive oxygen species (ROS) are considered to be important mediators of several biological responses, including cell proliferation and extracellular matrix deposition. Glucose auto-oxidation, non-enzymatic glycation, the formation of advanced glycation end products, and the overproduction of ROS by mitochondria are potential sources of hyperglycemia-induced oxidative stress [2,3]. Han and his collaborators demonstrated that high D-glucose concentrations (15, 20, and 25 mM) induced production of hydrogen peroxide and lipid hydroperoxide in PTCs cultured cells through dependent on mitochondrial ROS and NADPH oxidase [4]. Previous studies with cultured neuronal cells indicated that elevated glucose levels induce dysfunction and apoptosis, possibly through the formation of ROS [5].

However, several other reports have pointed out different results from experiments evaluating the adverse effect of glucose at high level of exposure. A report by Sakuma et al. indicated that high glucose inhibits apoptosis in coronary artery smooth muscle cells by up-regulating anti-apoptotic proteins [6]. Other studies using endothelial cells (EC) demonstrated that a high-glucose concentration induces proliferation effect and survival in human umbilical vein EC [7,8], human dermal microvascular EC [9], aortic EC [10], and retinal EC [11]. These conflicting studies may be explained, at least in part, by the differences in species, tissues of origin, or experimental conditions. Although there is enough scientific evidence showing the effects of glucose in normal cells, little is known about the molecular mechanisms of glucose overdose in cancer cells. Therefore, the present study was designed to test the cytotoxic, genotoxic, and apoptotic effects of D-glucose using human breast adenocarcinoma (MCF-7) cells as a test model.

## Materials and Methods

### Cell culture

The human breast adenocarcinoma (MCF-7) cells, purchased from the American Type Culture Collection -ATCC (Manassas, VA), were thawed by gentle agitation of their containers (vials) for 2 minutes in a water bath at 37°C. After thawing, the content of each vial was transferred to a 75 cm^2^ tissue culture flask, diluted with RPMI 1640 supplemented with 10% fetal bovine serum (FBS) and 1% penicillin and streptomycin, and incubated for 2 to 3 days at 37°C in a 5% CO_2_ incubator. The growth medium was changed twice weekly. Cells grown to 75–85% confluence were washed with phosphate buffer saline (PBS), trypsinized with 3 mL of 0.25% (v) trypsin-0.0.3% /v) EDTA, diluted with fresh medium, and counted using a hemacytometer.

### Analysis of cell viability by MTT assay

To determine cell viability following D-glucose treatment, 1 × 10^4^ cells were plated in each well of 96-well plates, and were placed in the humidified 5% CO_2_ incubator at 37°C to allow them to attach to the substratum for 2 to 3 days. The cells were exposed to different concentrations of D-glucose and placed in the humidified 5% CO_2_ incubator for 2 h. The cells incubated in culture medium alone served as a control for cell viability (untreated wells). Cell viability was determined using the MTT assay as previously described [12,13].

### Analysis of DNA damage by comet assay

The comet assay was carried out by the method previous described by Collins and his collaborators [14,15] with some modification [16]. Cells were counted (10,000 cells/well) and aliquots of 100 µL of the cell suspension were placed in each well of 96 plates, treated with 100µl aliquot of either media or D-glucose (0, 5, 10, 20, 40 and 80 mg/ mL) respectively and incubated in a 5% CO_2_ at 37°C for 2 h. After incubation, the cells were centrifuged, washed with PBS free calcium and magnesium, and re-suspended in 100 µL PBS. In a 2 mL tube, 50 µL of the cells suspension and 500 µL of melted LMAgarose were mixed and 75 µL was pipetted onto a pre-warmed cometslide. The side of the pipette tip was used to spread completely agarose/cells over the sample area. The slides were placed flat in the dark at 4°C for 10 minutes to allow the mixture to solidify and then immersed in prechilled lysis solution at 4°C for 40 minutes. The slides were removed from lysis solution, tapped, and immersed in Alkaline Solution for 40 minutes at room temperature in the dark. The slides were washed twice for 5 min with Tris-Borate-EDTA (TBE). The slides were electrophoresed at low voltage (300 mA, 25 V, 4°C) for 20 minutes. The slides were placed in 70% ethanol for 5 min, removed, tapped, and air-dried for overnight. The slides were stained with SYBR Green stain designed for the Comet Assay, and allowed to air dry at room temperature for six hours. SYBR Green stained cometslides were viewed with an Olympus fluorescence microscope and analyzed using LAI’s Comet Assay Analysis System software (Loates Associates, Inc. Westminster, MD).

### Analysis of apoptotic cells by flow cytometry

Apoptotic or necrotic cell levels were determined by flow cytometry after double staining with Annexin V-FITC and propidium iodide using an assay kit from BD Pharmingen as described previously [17]. Briefly, 2 mL of cells (1 × 10^6^ cells/mL) were added to each well of 12 plates and treated with 5, 10, 20, 40 and 80 mg/mL of D-glucose for 2 h. Control well plates were also made without D-glucose. After 2 h of incubation, 1 × 10^6^ cells/mL were counted and washed in PBS, re-suspended in binding buffer (10 mm Hepes/NaOH pH 7·4, 140 mm NaCl, 2·5 mm CaCl_2_), and stained with FITC-conjugated annexin V (Pharmingen, Becton Dickinson Co., San Diego, CA, USA). After staining, the cells were incubated for 15 min in the dark at room temperature. Cells were re-washed with binding buffer and analysed by flow cytometry (FACS Calibar; Becton-Dickinson) using Cell Quest software.

### Statistical analysis

Experiments were performed in triplicates. Data were represented as means ± SDs. Where appropriate, one-way ANOVA test or Student paired t-test was performed using SAS Software available in the Biostatistics Core Laboratory at Jackson State University. *P*-values less than 0.05 were considered statistically significant.

## Results

### Inhibition of cell viability

**Figure 1:**
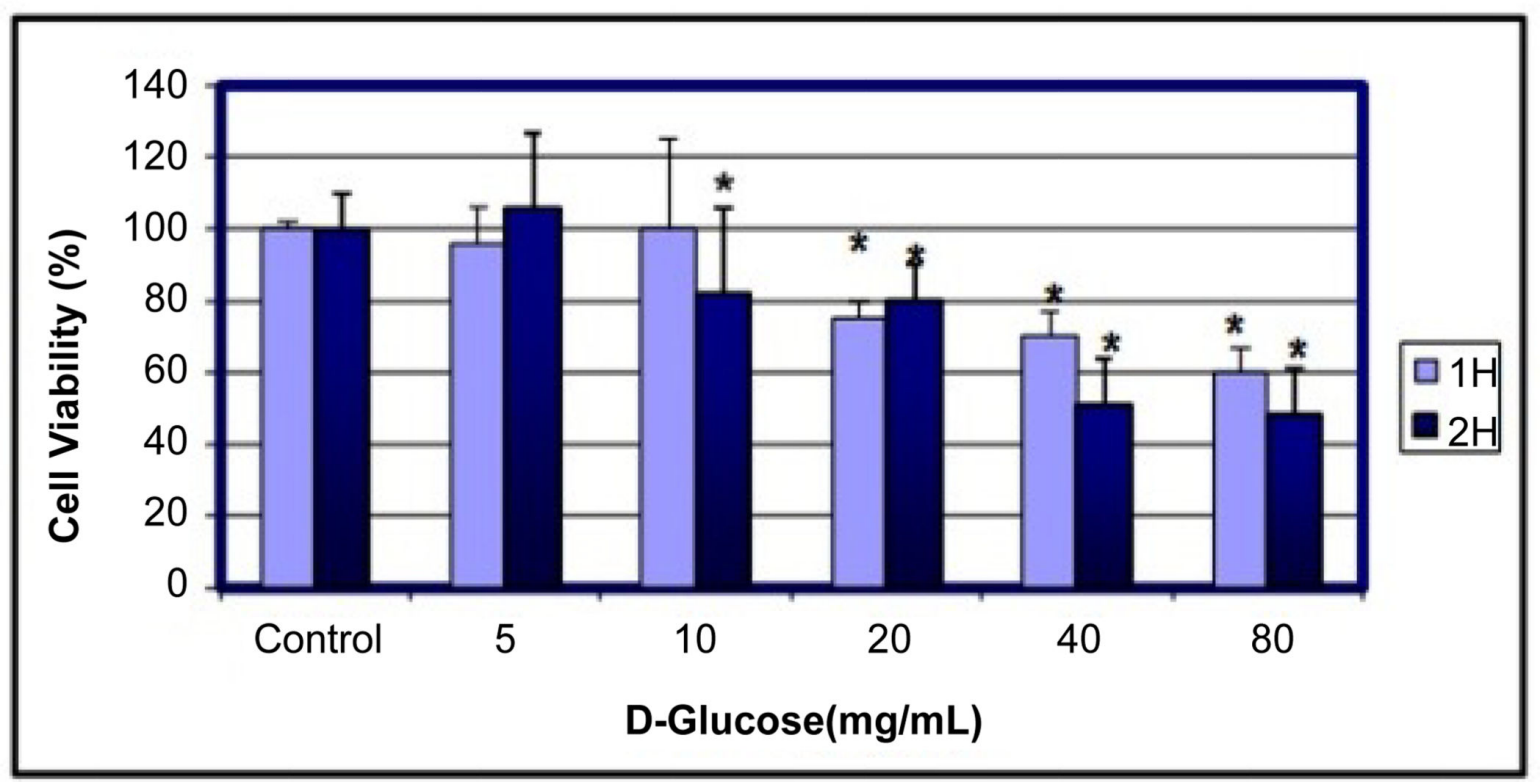
Cytotoxic effects of D-glucose to MCF-7 cells. Cells were cultured with different doses of D-glucose for 1 and 2 h as indicated in the Materials and Methods. Cell viability was determined based on the MTT assay. *Significantly different (p < 0.05) from the control, according to the Dunnett’s test.

Cells were treated with increasing concentrations of D-glucose for 1 h and 2 h, and viable cells were monitored by MTT [3–(4, 5-dimethylthiazol-2-yl)-2, 5-diphenyltetrazolium bromide] assay. Treatment of MCF-7 cells with 5, 10, 20, 40 and 80 mg/mL VA for 1 h decreased cell viability to approximately 4%, 0%, 25%, 30% and 40% respectively (Figure 1). Exposing MCF-7 cells for 2 h increased viable cell counts by 6% in 5 mg/mL D-glucose and decreased cell viability to approximately 18%, 20%, 49% and 52% in 10, 20, 40 and 80 mg/ mL D-glucose, respectively (Figure 1). Overall, data obtained from the MTT assay demonstrated that D-glucose treatment reduced cell viability in a concentration and time-dependent manner.

### Induction of DNA damage

**Figure 2:**
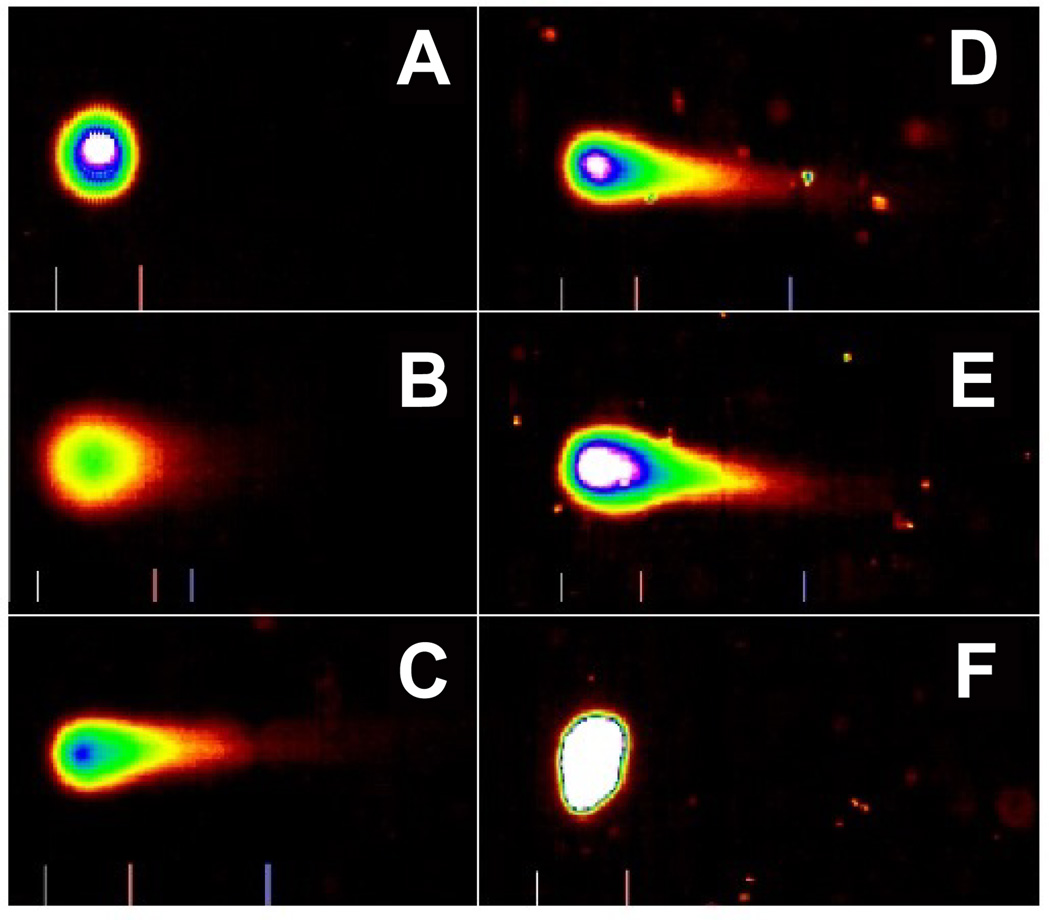
Representative SYBR Green Comet assay images of untreated (A-control) and D-glucose treated MCF-7 cells at 5 mg/mL (B), 10 mg/mL (C), 20 mg/mL (D), 40 mg/mL (C), and 80 mg/mL (F). High percentages (>60%) of selected images were observed in specific D-glucose dose, as indicated above.

Representative Comet assay images of control and D-glucose-treated cells using SYBR Green stain are presented in Figure 2. As denoted in this figure, there is gradual increase in the mean values of comet tail length, tail moment, and percentages of DNA cleavage in MCF-7 cells, with increasing doses of D-glucose. Overall, the results generated from the comet assay indicated D-glucose exposure is highly genotoxic to MCF-7 cells.

### Induction of apoptotic cells

**Figure 3:**
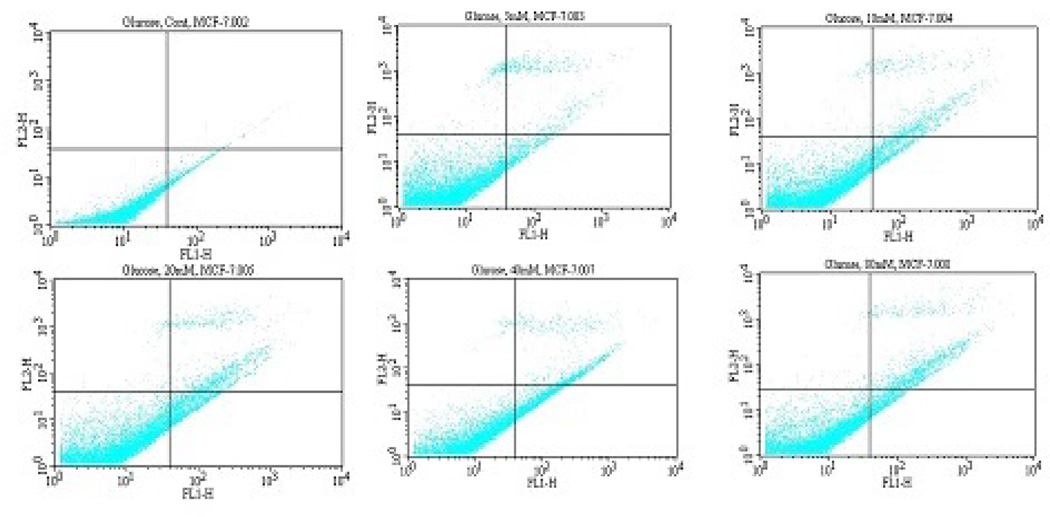
Representative dot plots showing the inhibitory effect of D-glucose to MCF-7 cells upon 2 h of exposure. A = Control Cell (Untreated), B = 5 mg/ mL D-glucose, C = 10 mg/mL D-glucose, D = 20 mg/mL D-glucose, E = 40 mg/mL D-glucose, F = 40 mg/mL D-glucose. 1 = Live cells (Annexin V^−^/PI^−^), 2 = Early apoptotic cells (Annexin V^+^/PI^−^), 3 = Late apoptotic or necrotic cells (Annexin V^+^/PI^+^), 4 = Necrotic cells (PI^+^).

Viable, early apoptotic, and late apoptotic or necrotic cells can be distinguished by flow cytometry analysis using dual staining with annexin V/PI dyes. Using this research approach, we treated MCF-7 cells with D-glucose for 2 h and measured the modulation of annevin V/PI negative and positive cells. We observed a strong dose-response relationship with regard to D-glucose exposure and annexin V positive cells (Figure 3). Viable cells were negative for both PI and annexin V; apoptotic cells were positive for annexin V and negative for PI, whereas late apoptotic or necrotic cells displayed both high annexin V and PI labeling. Non-viable cells undergoing necrosis were positive for PI and negative for annexin V. As shown on Figure 3, the percentages of annexin V/PI positive cells were: (3.5 ± 1.1.)%, (11.0 ± 0.5)%, (15.5 ± 2.3)%, (20.0 ± 5.1)%, (27.3 ± 6.4)%, and (21.2 ± 3.7)% in 0, 5, 10, 20, 40, and 80mg/mL D-glucose, respectively (Table 1).

## Discussion

The adverse effects of high glucose concentrations on various cancer cell types have been known for some time. Nevertheless, there remains considerable doubt about the cellular and molecular mechanisms of action of D-glucose in MCF-7 cells. In the present study, we first demonstrated that D-glucose exerts a concentration-dependent dual effect upon 2 h of exposure: it causes differentiation or proliferation of MCF-7 cells at low concentrations and cell death at relatively high concentrations. As seen in figure 1, D-glucose slightly increases the proliferation of cancer cells at physiologic or low concentrations (5 mg/mL) and gradually decreases the viability of cancer cells at high concentrations (10–80 mg/mL) in a dose and time-dependent manner.

Consistent with low or physiologic concentrations of glucose used in the present study upon 2 h, previous research indicated that glucose and other factors related to glucose metabolism including insulin and IGFs may contribute to breast cancer development [18]. Glucose may play a direct role in the development of breast cancer by favoring the selection of malignant cell clones [19]. For example, a study by Warburg showed that neoplastic cells use glucose for proliferation [19]. Increased metabolism of glucose toward the pentose phosphate pathways is one of the central metabolic characteristics of malignant tissues [20]. A large-scale epidemiological study of 21 modern countries that track morbidity and mortality (Europe, North America, Japan and others) revealed that sugar intake is a strong risk factor that contributes to higher breast cancer rates, particularly in older women [21].

Consistent with high concentrations (10–80 mg/mL) of glucose used in the present study, previous reports indicated that high glucose inhibit the proliferation, migration and **in vitro** angiogenic capacity of bone marrow-derived endothelial progenitor cells, and alter the regenerative potential of mesenchymal stem cells [22,23]. High glucose induced nuclear factor kappa B mediated inhibition of endothelial cell migration. In contrary to our finding, several other studies using endothelial cells (EC) demonstrated that high-glucose concentration increases cell growth and survival in human umbilical vein EC [7,8], human dermal microvascular EC [9], aortic EC [10], and retinal EC [11]. These conflicting studies may be explained, at least in part, by the differences in species, tissues of origin, or experimental conditions.

Next, we determined whether the glucotoxicity is associated DNA damage in MCF-7 cells; by the means of alkaline single cell gel electrophoresis (Comet) assay. We found that D-glucose has a strong genotoxic potential and is able of causing DNA damage in cancer cells. Our results demonstrated that D-glucose induces genotoxic effects to MCF-7 cells in a dose-dependent fashion, suggestive clear evidence that D-glucose may be a potent DNA damaging agent against breast cancer when used at high doses. There is limited scientific data in the literature explaining how D-glucose exposure induces DNA damage in cancer cells. Hence, further studies are needed to establish the genotoxic mechanism on the basis of the genetic damage induced by D-glucose. Thus, we showed that high glucose concentrations induce DNA damage in MCF-7 cells. Similarly, other studies in our laboratory showed that the size, shape and distribution of DNA within the comet correlate with the extent of DNA damage in human promyelocytic leukemia cells when treated with arsenic trioxide [24].

Furthermore, we examined whether D-glucose treatment induces apoptosis of MCF-7 cells. To accomplish this research objective, we performed Annexin V/propidium iodide (PI) staining that allows the discrimination of viable cells (Annexin V^−^/PI^−^), early apoptotic (Annexin V^+^/PI^−^), and late apoptotic or necrotic cells (Annexin V^+^/PI^+^). We observed that D-glucose induces apoptosis of MCF-7 cells (Figure 3). The obtained results show that the percentage of apoptotic cells was dependent on D-glucose concentration with maximal level of apoptotic cell death at 40 mg/mL (Table 1). In line with our current finding showing that high glucose induces apoptosis of MCF-7 cells, previous laboratory studies demonstrated that high glucose concentrations disturb cell cycle, induce DNA damage, delay endothelial cell replication, and cause excessive cell death in cultured human endothelial cells [25,26]. Other studies have pointed out that high glucose selectively triggers apoptosis in cultured endothelial cells [27,28]. In contrast to its effect on MCF-7 cells as seen in the present study, high glucose has been reported to inhibit apoptosis in coronary artery smooth muscle cells by up-regulating anti-apoptotic proteins [6]. Thus, the molecular events linking high glucose with the apoptotic machinery of cancer cells may be far more complicated than being realized. Cell death is thought to take place at least by two ways that include apoptosis and necrosis.

Apoptosis is an active and physiological mode of cell death that is believed to be mediated by active intrinsic mechanisms, although extrinsic factors can contribute [29–32]. Generally, it shows noninflammatory morphological changes of cell shrinkage which include cytoplasmic and nuclear condensation, chromatin condensation (pyknosis), ordered DNA fragmentation (karyorrhexis), blebbing of the plasma membrane, formation of apoptotic bodies (cellular fragmentation of membrane-bound fragments) and exposure of surface molecules such as phosphatidylserine (PS) on the plasma membrane to facilitate complete phagocytosis of apoptotic cells [33,34]. In contrast, necrosis is an uncontrolled cell death that is characterized by progressive loss of cytoplasmic membrane integrity, rapid influx of Na^+^, Ca^2+^, and water, resulting in cytoplasmic swelling and nuclear pyknosis [34–36]. The latter feature leads to cellular fragmentation and release of lysosomal and granular contents into the surrounding extracellular space, with subsequent inflammation [37,38]. A recent pharmacological study in our laboratory demonstrated that *Vernonia amygdalina* (Africa medicinal plant) induces growth arrest and apoptosis accompanied by secondary necrosis cell death of breast cancer (MCF-7) cells [39]. A 2010 scientific report demonstrated that the mechanism underlying glucose-induced β-cell death involves the upregulation of Fas receptors, which can interact with the constitutively expressed FasL on neighboring β-cells. Fas-FasL interaction leads to cleavage of procaspase-8 to caspase-8. Activated caspase-8, the most upstream caspase in the Fas apoptotic pathway, promotes caspase-3 activation and DNA fragmentation [40].

## Conclusion

We have demonstrated in the present *in vitro* study that relevant concentrations of D-glucose at low doses (5 mg/mL) slightly increase the proliferation of cancer cells upon 2 h of exposure while on the other hand, high doses (10–80 mg/mL) of D-glucose significantly (P < 0.05) reduce the viability of MCF-7 cells, induce DNA damage and cause apoptosis of MCF-7 cells with a maximum dose-dependent response at 40 mg/mL. Taking together, these data provide clear evidence that D-glucose overdose is a potential inducer of cytotoxic, genotoxic, and apoptotic effects in tumors cells such as MCF-7 cells. This finding represents the basis for further studies addressing the pathophysiological mechanisms of action of glucose overdose.

## Figures and Tables

**Table 1:** Summary data of annexin V assay obtained from the flow cytometry. Human breast adenocarcinoma (MCF-7) cells were cultured in the absence or presence of D-glucose for 2 h as indicated in the Materials and Methods. Values are shown as means ± SDs of 3 replicates per experiment.

Sample ID	Treatment	Annexin V/PI negative Cells or Live Cells (Mean ± SD)%	Annexin V/PI Positive Cells or Apoptotic cells (Mean ± SD)%
A	0 mg/mL	96.5 ± 1.1	3.5 ± 1.1
B	5 mg/mL	89.0 ± 0.5	11.0 ± 0.5
C	10 mg/mL	84.5 ± 2.3*	15.5 ± 2.3*
D	20 mg/mL	80.0 ± 5.1*	20.0 ± 5.1*
E	40 mg/mL	72.7 ± 6.4*	27.3 ± 6.4*
F	80 mg/mL	80.8 ± 3.7*	19.2 ± 3.7*

* *P* < 0.05 versus compared with control group.
